# Supplementary material for: Comparative whole-genome analyses of articular chondrocytes and skin fibroblasts reveal distinct genome instability landscapes in mesenchymal cell types
Source: PLoS Genet. 2026 May 20;22(5):e1012156. doi: 10.1371/journal.pgen.1012156 (PMC13211306; doi:10.1371/journal.pgen.1012156)
Supplement: Attachment — Submitted filename: Point_by_point_response_to_reviewers.pdf [file pgen.1012156.s023.pdf]

Point-by-point response to reviewer's comments.

We thank all reviewers for reading our manuscript and for providing useful suggestions.

In this point-by-point response letter:

- Original texts of reviewer's comments are shown in black font.
- Author's responses and quotes from the original submission are shown in blue font.
- New or modified text in the revised manuscript are shown in in red font.

Reviewer #1:

This manuscript by Sauty et al., conducted whole-genome sequencing of human chondrocytes and compared the mutational landscape with skin fibroblasts. Mutational signature and motif-centered analyses indicate that chondrocytes mainly include endogenously accumulated mutations associated with aging. In comparison, skin fibroblasts show strong mutational patterns induced by UV radiation. Overall, this is an interesting report comparing somatic mutations between two types of skin cells. The data interpretation is clear and the underlying mechanisms are discussed reasonably well.

My only comment is related to chondrocytes collected from donors with osteoarthritis (OA). The authors mentioned there is little difference between healthy chondrocytes and OA-chondrocytes. But they may have missed an opportunity to identify mutation sites in important genes or gene regulatory elements associated with OA. Adding additional mutation analysis to identify potential OA-associated gene mutations may increase the significance of this study.

We thank the reviewer for positive evaluation of our work and for useful suggestion. We annotated all mutations in chondrocyte clones and single cells for functional impact, cancer prediction, and coincidence on OA effector gene. We have created a new Results section in lines 559-572 titled **Functional annotation of somatic SNVs and InDels detected in chondrocytes** and provided the annotation information in supplementary table S7.

Reviewer #2:

In the presented manuscript, Sauty and colleagues describe their study of mutations in chondrocytes. The authors utilize cloning of single chondrocytes to investigate mutations in individual cells from nine donors. Based on the resulting mutation sets, they analyze mutation burden and spectra, mutational motifs, and structural variations. To expand the dataset, they also incorporate mutations identified in prior single-cell studies of chondrocytes from more than a dozen additional donors (ref. 21).

Several donors with osteoarthritis (OA) were included both in this and in the previous study. Somewhat surprisingly, both studies report minimal differences between mutational patterns in chondrocytes derived from OA versus normal donors.

The authors further compare the mutational landscapes of chondrocytes and skin fibroblasts. Although both cell types originate from the mesoderm, they reside in distinct anatomical environments and are subject to different endogenous processes and environmental exposures. Consistent with this, the authors identify multiple differences between fibroblasts and

chondrocytes, including:
* Distinct mutational burdens and spectra for both SNVs and indels
* Greater influence of UV-induced damage in fibroblasts
* Stronger contribution of endogenous mutagenesis in chondrocytes
* Differences in the distribution of structural variations across genomic regions

The study leverages both signature-based and motif-based analyses of mutations, which make the analyses well rounded. I would like to emphasize this as a particular strength of the work. Mutational signature analysis is a broadly applicable approach that can identify patterns reflecting underlying mutational processes without prior assumptions. However, it operates in a 96-dimensional space (96 features), and the large (more than 96) number of known signatures complicate decomposition, leading either to contamination by multiple signatures or failure to detect biologically relevant ones.

In contrast, motif-based analysis is less general—since it relies on prior knowledge of sequence context—but offers greater robustness in quantifying contributions from known mutational processes. This complementarity is well illustrated in both the authors' previous work and the current study. For example, signature-based analysis detected multiple signatures across clones and cells, yet SBS1 was identified only in chondrocyte clones and was missed in other datasets (Figure 2A). Given that SBS1 reflects spontaneous deamination of methylated cytosines and is expected to be ubiquitous across human cell types, this result is somewhat unexpected. Motif-based analysis, however, confirms its presence across datasets (Figure 3). Notably, the previous study of chondrocytes (ref. 21) also failed to detect SBS1 using signature-based approaches.

These observations underscore how the two analytical frameworks complement each other, and it is commendable that the authors applied both. Importantly, motif-based analysis also enabled a novel insight: chondrocytes appear to be more strongly influenced by endogenous mutagenic processes than fibroblasts. The authors also appropriately address potential overlap between motifs associated with different mutational processes.

We thank reviewer for detailed analysis of our manuscript and for acknowledging the relevance and value of our motif-centered analysis.

Overall, I find the analyses presented in this manuscript compelling. However, I would like the authors to address the following points prior to publication:
* In general, comparisons of mutational burden (SNVs, indels, and SVs) between single cells and clones are not particularly informative. While it is reasonable to expect that fibroblasts from sun-exposed skin carry higher mutation loads than chondrocytes, more subtle differences—such as those shown in Figure 6A between cloned and single chondrocytes—may be influenced by technical artifacts. The experimental approaches and variant-calling methods differ substantially between single-cell and clonal datasets. I suggest moving such comparisons to the Supplementary Materials.

We thank the reviewer for this comment. We agree that the differences in mutational burden between cloned and single chondrocytes are subtle and could be influenced by technical variation between the two methods. However, we believe such comparisons are essential for the main text and display items as they establish the baseline mutation counts used for all

subsequent granular analyses and ensure transparency regarding our raw data. To address the reviewer's concern, we have added a statement acknowledging that technical artifacts may contribute to the subtle differences observed between these groups. Lines 499-502 now read:

While we observed a lower number of indels in single chondrocytes compared to chondrocyte clones (Fig 6A), we note that this difference may be influenced by the distinct variant-calling sensitivities and technical artefacts inherent to both single-cell and clone sequencing.

* Figure S2: Given that mutations were selected within a narrow VAF range (45% < VAF < 55%), mutation burden estimates for clones A06, A08, and A11 may be substantially biased because of peak in VAF distribution is not at 50%. The authors should comment on the potential biased mutation calling for those clones or consider removing them from the analyses.

We acknowledge that we are reporting a conservative estimate of the total mutation burdens in these clones. We opted for this stringent VAF filtering criteria to ensure we are working with clone-specific mutations. While this may influence the comparison of total mutation burdens, we want to highlight that these clones remain valid for motif enrichment analyses central to the conclusions of this manuscript.

Enrichment of mutational motifs is calculated as a ratio of two ratios: fraction of the focal mutation in a motif to all mutations, and the fraction of the motif to all bases. Calculations of both sample-specific and donor-specific enrichment values are mathematically independent of the absolute mutation count as long as the mutation calls with different VAFs are true somatic mutations. In the initial submission, we provided support for the validity of utilization of these clones in S6A Fig, and S5 table, and in lines 314-319 where we detect the presence of enrichment-adjusted mutations in the two ubiquitous motifs regardless of a VAF fraction used for mutation call filtering:

Quote (original text in blue font, added text in red font):
"We also performed motif-centered analysis with mutations from different allele fractions detected in the chondrocyte clones sequenced in this study. We detected the two ubiquitous motifs aTn and nCg in 100% of donors in low (<45%), stringent (45%-55% or ≥90%), and no (20%-100%) allele fraction filters (S6A Fig, S5a table). This verified that the low allele fraction mutations in chondrocyte clones are not *in vitro* culture induced, but rather subclonal in nature (i.e. possibly arisen from partial fusion with a neighboring colony).

* Lines 283–285: The term "subclonal nature" is unclear in this context and should be explicitly defined.

In line 319, we clarified "subclonal nature" with "i.e. possibly arisen from partial fusion with a neighboring colony"

* Lines 363–365: The definition of the enrichment metric is difficult to follow. It would be helpful to move this explanation to the Methods section and/or provide a schematic, equation, or supplementary figure for clarity.

We have moved updated definition of the enrichment metric to the Methods section and added "see Methods" in line 401. In lines 880-889 in Methods, we added a simplified equation for enrichment calculation. The text in Methods section now reads:

Enrichment is calculated as the ratio of two ratios: the ratio of mutations in motif to all mutations, and the ratio of the number of motifs to all bases in genomic context of ±20 bases surrounding mutations ("Genome" in the below formula). For this calculation, context is defined as the +/- 20 bases surrounding the mutated base. The use of genomic context immediately surrounding the mutated nucleotide rather than the whole genome sequence helped to concentrate on the sections of the genome that were actually sequenced and on localized preference of mutagenesis stemming from small-range preference of lesion occurrence, and to alleviate confounding effects of other factors, including epigenomic features influencing overall mutagenesis within greater genomic regions. This is shown in a simplified formula below.

$$Enrichment = \left(\frac{Mutations\ in\ Motif}{All\ Mutations}\right) : \left(\frac{Motifs\ in\ Genome}{Bases\ in\ Genome}\right)$$

* Across multiple figures, axis ranges (e.g., for age, enrichment, and mutation burden) vary substantially, making comparisons between panels difficult. For example, in Figure 1B, the y-axis for chondrocytes ranges from 100–650 in one panel and 200–800 in another, while the x-axis ranges from 55–90 and 30–90. Such inconsistencies hinder interpretation. I recommend standardizing axis scales within panels for the following figures: 1B, 5A–C, 6B–D, 7B, S3B, S7A–C, and S10B.

We thank the reviewer for this suggestion. The primary objective of the scatter plots in figures 1B, 5A–C, 6B–D, 7B, S3B, S8A–C (previously S7A–C), and S12B (previously S10B) is to illustrate within-group trends and the distribution of data points for individual cell types. Using a standardized scale for all will obscure data points and show misleading trendlines. We request the reviewer refer to the box and bar plots in figures 1A, 4, 6A, 7A, S3A, S12A for direct comparison between cell types. For further clarity, we have now added the following statement in the legends of Figure 1B, 5A–C, 6B–D, 7B, S3B, S8A–C, and S12B:

Axis ranges for scatter plots in panel X were scaled to the specific range of data points within each cell type in order to maximize the resolution of internal trends and data distributions. For direct quantitative comparisons between groups using standardized scales, refer to the corresponding box plots in Fig Y.

Reviewer #3:

Sauty et al. sequenced single-cell-derived chondrocyte colonies from healthy individuals and those suffering from osteoarthritis and provide analysis of somatic base substitution, indel and structural variants. Their data were supplemented by previously published whole genome-amplified single chondrocytes - which served a confirmatory role - and skin fibroblasts - which served as a contrasting mesodermal cell type. These data address questions about what mutagenic processes are active in healthy and diseased human tissues, an area of active research that has accelerated in recent years due to technological advances. The authors' study is both a timely and valuable contribution to these ongoing efforts.

Comparison of the three datasets revealed no significant difference between healthy and osteoarthritic chondrocytes. It did, however, point toward differences in the types and rates of mutations accumulated by chondrocytes and fibroblasts, and these differences were consistent with the proliferative capacities and exposures to exogenous mutagens of the two cell types. The authors' preferred approach, motif-centered analysis, differs from the more widely-used signature analysis and allows some mutagenic processes to be more directly probed.

While most of the conclusions are very sensible, some are troubling. For example, none of the motifs related to endogenous mutagenesis were correlated with donor age in clonally expanded chondrocytes, despite the main message of the work being that these same endogenous processes are the active ones in these cell types. Instead, this claim was substantiated only by the previously published single-cell amplified chondrocytes. I suspect this may be related to a lack of normalization between the 3 datasets compared in this study, which is itself a considerable challenge due to the very different technical approaches taken. However, these concerns can be reasonably addressed through a (hopefully not too burdensome) few changes to the analysis.

We thank the reviewer for critical analysis of our results and for useful suggestions.

Major concerns

1. The authors compare their own single cell colony mutation counts to those from a single-cell study and find similar counts (Figure 1A-B). I find this surprising since there appears to be no correction for sensitivity for either the authors' own data or the SCMDA data. Given the technological differences I would expect sensitivity to be fairly different, especially given that SCMDA's sensitivity has been estimated between 40-50% (PMID 31467286).

We thank the reviewer for this comment. We would like to clarify that in Figure 1A-B, we are presenting the raw mutation counts for both clones and single cells that were used for all subsequent analyses to show the true breadth of analyzed mutations.

To ensure detection of clone-specific mutations, we filtered mutations in chondrocyte clones within a strict VAF range (45%–55% for heterozygous and ≥90% for homozygous variants). This method for identifying high-confidence somatic mutations in single-cell-derived clones has been validated in our previous study on skin fibroblasts by performing PCR amplification and Sanger sequencing on identified mutations which showed ~100% detection sensitivity (PMID 27788131). However, many mutations are also detected in clones at low VAFs that are not retained in the final call set. SCMDA on the other hand suffers from low sensitivity and amplification bias. These different technological constraints limit our ability to draw definitive conclusions about the absolute difference in total mutation burden between chondrocytes clones and single cells. However, our primary findings rely on the calculation of enrichment of mutational motifs, which is not influenced by the sensitivity of mutation detection.

To provide full transparency, we have added Supplementary Table S2f, which reports the mutation counts for chondrocyte clones before and after allele fraction filtering, alongside the observed and estimated counts for single cells reported in the source study. Line 161-164 now reads:

The raw counts of mutations detected in chondrocyte clones prior to allele fraction filtering, as well as the expected number of mutations in single cells after sensitivity correction, are reported

in S2f table along with the final mutation counts for both groups used for all subsequent analyses.

2. The finding that healthy chondrocytes had higher burdens than OA chondrocytes is surprising. However, to make this claim the authors must account for differences in age distributions between the OA and healthy cohorts. This could be done by performing statistics on the residuals from the linear regressions against age. This would also benefit from correction for sensitivity since sensitivity between single cells can be quite variable due to the nature of the amplification.

We thank the reviewer for this suggestion.

To address this, we regressed total mutation burden against age using a linear mixed-effects (LME) model, including donor as a random effect. We then performed a Mann-Whitney test on the residuals to assess differences between healthy and OA groups independent of age and did not observe a statistically significant difference in either the chondrocyte clone or single-cell datasets.

We note that this finding differs from that reported by Ren et al. (2025), who observed higher sensitivity-corrected mutation burdens in healthy donors after explicitly accounting for technical sensitivity and age in LME models and leave-one-out studies. This discrepancy likely reflects the impact of sensitivity correction on single cell data. At the same time, it is important to note that while single-cell sequencing captures the full spectrum of the chondrocyte population in vivo, clonal expansion may select against heavily mutated or senescent cells that are present in vivo in the tissue but incapable of forming colonies in vitro. Consequently, the absence of a significant difference in mutation loads and patterns between healthy and OA clones may reflect a survivals bias toward high-fitness, low-burden progenitors across both healthy and OA tissues. We again wish to reiterate that the calculation of mutational motif enrichment is not impacted by platform-specific variables and is therefore more suitable than total mutation burden to compare samples sequenced using different methodologies.

We have now created a supplementary figure panel S3D plotting the residuals and showing p-values from Mann-Whitney U test. Model summaries are provided in S3g table. In the results section, we have summarized this analysis in lines 186-197.

To evaluate whether differences in mutation burden between chondrocyte groups were influenced by the varying age distributions of the cohorts, we modeled mutation burden as a function of age and donor identity using a Linear Mixed-Effects (LME) model treating the donor as a random effect to account for inter-individual variability. We compared the resulting residuals between chondrocyte healthy and OA groups using a Mann-Whitney U test. Our analysis of raw mutation counts did not reveal a statistically significant difference in age-adjusted burdens between healthy and OA donors in either the clone or single-cell dataset (S3D Fig, S2g table). This is in contrast to the previously reported high mutation burden in healthy chondrocyte single-cell donors after correcting for SCMDA mutation detection sensitivity, suggesting that the higher burden may depend strongly on mathematical sensitivity correction or be restricted to a specific subpopulation of cells not captured by clonal propagation.

We have also updated the figure legend and methods section respectively.

3. Continuing the sensitivity point: another reason the authors should correct for sensitivity is so their aging rates for chondrocytes are compatible with the growing literature on aging rates. Their reported counts of ~300 substitutions for a 50-year-old is far below expectation, with even non-proliferating cell types gaining ~20 substitutions per year (=1000 substitutions at age 50, PMID 33911282). Correcting for sensitivity might also improve the linear regression fit.

We believe that we have already addressed this point in response to Major concern 1. Briefly, the final number of mutations analyzed in this study is not the same as the absolute number of mutations detected in chondrocyte clones, or the sensitivity-corrected mutation counts in chondrocyte single cells. The absolute number of mutations in chondrocyte clones were filtered for stringent VAF, while the absolute number of mutations in single cells was not corrected for sensitivity. As shown now in S2f table (detailed in response to Major concern 1), the absolute count of mutations detected in chondrocyte clones before VAF filtering, as well as the sensitivity-corrected mutation load in single cell is much higher than in the final sets of mutation calls used in motif-centered analysis. PMID 33911282 example mentioned in the reviewer's comment used yet another sequencing technology, Nanopore TwinStrand (duplex sequencing) of DNA from bulk tissue samples. The absolute numbers of mutations reported across these different platforms are largely unsuitable for direct comparison as they have different detection efficiencies and sequence different biological materials that are likely representing different cell populations.

4. It is unclear how accurately cell division count can be predicted by the telomere length estimates. The authors should provide a supplementary figure showing telomere length estimates vs. age to give a sense of how variable the estimates are. Additionally, SBS1 - one of the clock-like substitution signatures - is thought to be indicative of cell division. The authors could support their findings by showing a correlation between SBS1 burden and their telomere-based division estimates.

We thank the reviewer for this suggestion. Supplementary figure S3E now shows the average estimated telomere length in the progenitor cell per donor plotted against donor age. Spearman correlation analyses showed significant positive correlation between donor age and telomere length in chondrocyte clones, while fibroblasts showed no correlation. This likely reflects survival bias of the high-fitness progenitor cells, especially for chondrocytes that tend to be quiescent and are further supported by comparable mutation loads in healthy and OA donors.

Because we did not perform agnostic signature extraction, we performed spearman correlation analyses between average nCg MEML per donor (analogous to SBS1 signature associated with spontaneous deamination of meCpG) and estimated number of cell divisions now shown in supplementary figure S3F. We did not find any significant correlation between estimated cell division rate and nCg MEML in either chondrocyte or fibroblast clones. This lack of correlation suggests decoupling of the two biological clocks. While nCg MEML reflects accumulation of mutation over chronological time and is influenced by both DNA replication and damage repair efficiencies, telomere-based estimate reflects only the replicative history of the progenitor cell. Together, our data capture the mutagenic profile of a sub-population of cells that have retained colony-forming capacity in vitro.

We have updated the results section in lines 220-236 as follows:

We performed spearman correlation analyses between donor age and estimated telomere length in the cells and detected significant positive correlation between donor age and telomere length in chondrocyte clones, while fibroblasts showed no correlation (S3E Fig). This likely reflects survival bias of the high-fitness progenitor cells capable of *in vitro* colony formation, especially for chondrocyte cells that tend to be quiescent. Short, eroded telomeres act as a critical checkpoint barrier that prevents quiescent cells from re-entering the cell cycle, often triggering senescence or apoptosis (35). This survival bias is further supported by comparable mutation loads in chondrocyte clones from healthy and OA donors (S3A Fig). We further tested if the accumulation of clock-like mutations correlates with replicative history of the progenitor cells. We performed spearman correlation analysis between telomere-derived cell division estimates and the average mutation load associated with spontaneous deamination of meCpG (C→T mutations in nCg motif, see later sections for explanation) (S3F Fig.). We found no significant correlation between cell division rates and meCpG-associated mutations in either cell type. This independence suggests that mutations in meCpG and replicative attrition of telomeres represent two distinct and decoupled facets reflecting aging of cells that have formed each single-cell colony. Taken together, these data indicate that our single-cell cloning methodology selectively captures a high-fitness cell sub-population.

In the discussion in lines 734-736, we have added:

Together, these two sets of data and analyses argue against an increased mutational burden in OA chondrocytes. One explanation is that there was selective survival and/or colony expansion of OA chondrocytes with more modest burden as compared to other chondrocytes from the same tissue that were not captured in sequencing. This interpretation of survival bias is further supported by the telomere analyses, which showed capturing of progenitor cells with longer telomeres from older donors regardless of OA status.

Figure legends have been updated accordingly.

5. How do the authors explain that the hTg motif was found in all single cell chondrocyte categories but none of the clonally expanded chondrocytes? Could it be a single-cell sequencing artifact?

We thank the reviewer for this observation. While donors with hTg motifs are detected across all single chondrocyte cell groups, the actual number of samples with detectable hTg MEML is very low (11 out of 100 single chondrocyte samples). As mentioned in line 358 in results, hTg motif is associated with exposure to S_N2 alkylating agent MMS, as opposed to aTn motif which serves as a probe for a broader class of S_N2 reacting electrophiles and is detected across all donors and cell types. It is possible that specific alkylation events (hTg) are stochastic and less likely to be represented in a selectively expanded clonal population, as opposed to the ubiquitous motif aTn that is more readily detected.

We have now provided S5g table that reports sample-specific MEML values for all motifs in all cell types analyzed in this study. Lines 358-365 in results now reads:

We also detected a low percentage of sun-exposed skin fibroblast and single cell chondrocyte donors with prevalence of hTg motif associated with exposure to S_N2-type alkylating agent Methyl methanesulfonate (MMS) (43), however the number of samples with hTg MEML was very low and potentially representative of stochastic alkylation events in a heterogenous single cell population (Fig 3, S5g table). Together, these findings suggest that the mutagenic activities attributable to small-epoxide and broader S_N2 electrophile exposure are detectable in both chondrocytes and skin fibroblasts and are more enriched in chondrocytes.

6. Is there really a lack of correlation (the P-value is 0.036) between nCg MEML and rCg enrichment in fibroblasts (Figure 5B?). There seems to be a very nice linear relationship between almost all points except 2-3 at the bottom right. This would seem to be consistent with the authors' interpretation since rCg does not overlap SBS7b (yCg).

We agree with the reviewer and have updated the text in lines 391-394 as follows:

We detected strong positive correlation of nCg MEML with both rCg MEML and yCg MEML in chondrocytes, as opposed to skin fibroblasts which only showed significant positive correlation with rCg MEML but not yCg MEML potentially confounded by UV-mutagenesis (Fig 5B, C, S5d table).

7. Line 379-380: am I understanding correctly that the authors are stating the rightmost 2 bars in the top right of Figure 4A are similar? Perhaps the statistics are not significant when comparing the groups, but that likely is only reflecting the n=2 sun-exposed fibroblasts rather than evidence that the enrichments are similar.

We agree with the reviewer. The text in lines 415-417 (previously 379-380) now reads

There was no statistically significant difference in the enrichment of yCn and nTt MEML between sun-protected and sun-exposed skin fibroblasts, however, sun-exposed skin fibroblasts showed significantly higher yCn and nTt MEML (Fig 4A, B, S5b table).

8. It is very concerning that motif-age correlations were found only in the previously published single-cell chrondrocyte data and not supported by the authors' own clonally expanded chondrocytes. The explanation for this (lines 428-430) would be easier to accept if some more analysis was shown - e.g., at least a supplementary figure with line plots showing why the correlation coefficients are insignificant for the motifs in clonally expanded chondrocytes.

We have included a new Supplementary Figure S9 illustrating the distribution of clock-like motif MEML against donor age for both chondrocyte clones and single-cell cohorts (S9C-D). To determine if the lack of correlation in our clonal dataset was a result of the restricted donor age range (55–86 years), we down-sampled the single-cell cohort (n=17) to include only 10 donors falling within that same age window as the chondrocyte clone donors and evaluated correlation with donor age (S9E). We found that the significant age correlation previously detected in the full single-cell cohort was lost within this narrow age range, suggesting that the lack of correlation in our clone dataset is likely due to the limited age distribution of the donors. The text in lines 475-484 now reads:

While both clock-like motifs aTn and nCg show significant age correlation in the single chondrocyte cohort, such tendency was not detected in cohort with clonally expanded chondrocyte samples (S10C, D Fig). To assess if the lack of correlation in our dataset of chondrocyte clones could be a result of the restricted donor age range (55–86 years), we down-sampled the single-cell cohort (n=17) to include only 10 donors falling within that same age window as the chondrocyte clone donors and evaluated correlation with donor age. We found that the significant age correlation previously detected in the full single-cell cohort was lost

within this narrow age range (S10E Fig). This suggests that the lack of age correlation with aTn and nCg MEML in chondrocyte clones are likely attributable to the small sample size, narrow donor age distribution, or positive selection of clones that evaded damage-induced apoptosis.

9. I was very surprised to find after reading the main text that the data were a mixture of two very different sequencing platforms: NovaSeq and DNBSEQ. Different sequencing technologies have different biases, and these artifact modes are often specific to exactly the sort of trinucleotide motifs analyzed in this work. The authors should (1) provide a table describing which technology was used for which samples (apologies if there is already such a table, I may have overlooked it) and (2) provide some analysis showing that NovaSeq and DNBSEQ provide similar signatures/motif enrichments. (2) need not be an exhaustive re-analysis of everything in the manuscript, but some reasonable confidence must be built.

We acknowledge the reviewer's concern regarding potential platform-specific biases between NovaSeq and DNBSEQ technologies. We want to highlight that the calculation of mutational motif enrichment and enrichment-adjusted MEML is localized to ±20nt window around the mutation which facilitates bypassing any hypothetic sequencing bias introduced by different representation of genomic regions by different sequencing instruments. We also would like to clarify that libraries for all samples were prepared using the same kit (Watchmaker DNA Library Prep Kit with Fragmentation (Watchmaker Genomics)) regardless of the instrument used for generating reads.

We have now updated Table S1 to include the sequencing platform used for each chondrocyte sample sequenced in this study. The same library preparation kit used for all samples has been already indicated in the Methods section of the initial submission, now in line number 791.

As per the reviewer's suggestion, we conducted comparisons of key metrics between the two platforms using two-tailed Mann-Whitney U tests. We found no significant differences in average sequencing coverage (p value 0.1011), total SBS burden (p value 0.5148), or the enrichment-adjusted MEML of aTn and nCg motifs (p values 0.4457 and 0.9483 respectively). These results show that the choice of sequencing platform did not introduce detectable artifacts into our mutational motif analysis.

We added the following statement in lines 818-822 of the Methods section:

Because data were generated using both NovaSeq and DNBSEQ platforms, we assessed potential batch effects by comparing key metrics between the two platforms using two-tailed Mann-Whitney U tests. We found no significant differences in average sequencing coverage (p value 0.1011), total SBS burden (p value 0.5148), or the enrichment-adjusted MEML of aTn and nCg motifs (p values 0.4457 and 0.9483 respectively).

Minor comments

1. The authors might consider using the standard colors for C>A (blue), C>G (black), C>T (red), etc. base changes as popularized by COSMIC. This could make it easier for readers familiar with the literature.

We have updated Figure 1 with standard colors for base changes as utilized by COSMIC.

2. This is not the topic of this work, so only a minor point: the authors show a large increase in T>G, in sun-exposed fibroblasts, yet UV light exposure signatures feature C>T, T>A and T>C (signatures SBS7a-b, SBS7c and SBS7d from COSMIC, respectively). The authors comment that the T>Gs are hard to assign to a mutagenic source, so what could explain them? They don't seem to be technical artifacts since they do not occur in the new clonally expanded chondrocytes.

We thank the reviewer for this thought-provoking question that, as outlined below, led us to detect a minor motif associated with UV mutagenesis. Given that this pattern was only visible in skin fibroblasts, we ran motif-centered analysis on yTn motif designed around cT and tT potentially mutagenic photoproducts formed by UV instead of the more mutagenic C-centered CPDs formed in yCn motif. We looked for all base substitutions in the yTn motif and found T>G to be the most prominently detected mutation. Spearman correlation analyses showed significant correlation of both UV motifs yCn→yTn and nTt→nCt with yTn→yGn MEML, which is now shown in Fig S9A-B. While the number of yTn→yGn MEML is ~100-fold less than yCn→yTn MEML, we speculate this may be a yet unidentified minor UV-associated motif.

In the Results section reporting strand bias, we have added lines 253-257:
Insertion of cytosine across TT photoproducts by Rev1 is extremely inefficient *in vitro*, however, its structural role in recruiting the REV1-Pol ζ complex is essential for the mutagenic bypass of UV-induced lesions (40, 41). This process may result in sporadic incorporation of a cytosine opposite a damaged thymine, which is subsequently fixed as a T>G transversion during the next round of DNA replication.

In the Results section reporting mutational motifs, we have added lines 421-430:

To investigate the significant enrichment of T>G mutations on the non-transcribed strand of only skin fibroblasts (Fig 1C), we further analyzed enrichment of mutations in yTn motif which encompasses CT and TT dimers. We detected multiple fibroblast samples with significant enrichment of yTn→yGn motif (S5g table). Spearman correlation analyses showed significant correlation of both UV motifs yCn→yTn and nTt→nCt with yTn→yGn MEML, more prominently in sun-exposed skin fibroblast samples (S10A, B). These results indicate that the observed T>G strand bias in skin fibroblasts may be driven by the mutagenic bypass of persistent UV-induced pyrimidine dimers containing thymines on the non-transcribed strand, likely through a REV1-mediated translesion synthesis mechanism that favors cytosine insertion opposite damaged thymines.

3. Related to (2), the axis break in Figure 1A completely hides the T>A burdens in sun-exposed fibroblasts and makes it hard to tell how much T>C and C>T there is. Perhaps the authors could make this clearer?

We have changed the scale break on Figure 1A to make all base changes visible within all cell types.

4. It would be helpful to present the substitution spectra for the 7 cell type/technologies in Figure 2A.

We have created a new supplementary figure S4 showing spectra for the 7 cell types shown in Figure 2A.

5. Line 478: what are "homonucleotide runs of 1bp repeat units"? Does this mean a single base deletion not in a homopolymer?

We recognize confusion in terminology of indels in long and very short homopolymers. We revised the text on lines 528-537 as follows.

"We found single base indels of thymine (and adenine in reverse complement) in homonucleotide runs consisting of ≥5 repeated single-nucleotide units to be the largest contributor in the chondrocyte clone indel matrix (deletions 17%, insertions 12% of all indels detected in chondrocyte clone samples) (S6f table). Chondrocyte single cells also showed similar preference to insertion of thymine (and adenine in reverse complement) in homonucleotide runs consisting of ≥5 repeated single-nucleotide units as the highest contributor (25% of all indels detected in chondrocyte single cell samples). Unlike chondrocytes, single base deletions in dinucleotide homopolymers (10% in di-cytosine (and di-guanine in reverse complement), 7% in di-thymine (and di-adenine in reverse complement)) constituted the highest fractions of indels associated with homonucleotide runs in skin fibroblasts (S6f table)."
